# 74. Maternal Dolutegravir (DTG) Use During Pregnancy and Birth Outcomes: The Antiretroviral Pregnancy Registry (APR)

**DOI:** 10.1093/ofid/ofab466.074

**Published:** 2021-12-04

**Authors:** Vani Vannappagari, Jessica Albano, Leigh Ragone, Taylor Cook, Angela Scheuerle, William R Short, Claire Thorne, Karen P Beckerman, Nahida Chaktoura, Lynne Mofenson

**Affiliations:** 1 ViiV Healthcare, Research Triangle Park, NC; 2 Syneos Health, Wilmington, North Carolina; 3 University of Texas Southwestern Medical Center, Dallas, Texas; 4 University of Pennsylvania, PA; 5 University College London Great Ormond Street Institute of Child Health, London, England, United Kingdom; 6 Staten Island University Medical Center, New Rochelle, New York; 7 NICHD, Bethesda, MD; 8 Elizabeth Glaser Pediatric AIDS Foundation, Silver Spring, Maryland

## Abstract

**Background:**

The APR is prospective exposure-registration cohort study, monitoring for early warning signals of major teratogenic effects of antiretrovirals (ARV) used during pregnancy. This analysis aimed to assess maternal demographics, pregnancy and neonatal outcomes including birth defects among infants with periconception and prenatal exposure to DTG using APR data.

**Methods:**

Descriptive analysis with frequency tabulation of pregnancy and neonatal outcomes is reported. Periconception is defined as any exposure within two weeks prior to or through 28 days after conception.

**Results:**

There were 1010 prospectively reported pregnancies with exposure to DTG through 31January2021, with 526 periconception exposures, 105 exposed later during 1^st^ trimester, 260 during 2^nd^ trimester and 119 during 3^rd^ trimester. Maternal median age at conception was 30 years and 77.0% of pregnancies were reported from the United States. At the time of reporting, 46.6% had CD4 count ≥500 cells/µL, 31.8% had 200-499 cells/µL, 12.5% had < 200 cells/µL and 9.1% unknown.

The 1010 DTG exposed pregnancies resulted in 1036 outcomes: 956 (92.3%) live births (26 twin pairs), 12 (1.2%) stillbirths, 28 (2.7%) induced abortions, and 38 (3.7%) spontaneous abortions. Among live births, 39 (4.1%) reported birth defects. For 1^st^ trimester exposures, overall defect prevalence was 3.3% (19/576, 95% CI:2.0-5.1) and for 2^nd^/3^rd^ trimester exposures defect prevalence was 5.3% (20/380, 95% CI:3.2-8.0). One neural tube defect (NTD) case of anencephaly with periconception DTG exposure was reported.

Among the 873 singleton, live births without birth defects, 92 (10.5%) were preterm (< 37 weeks of gestation); 103 (11.8%) had low birth weight (lbw) < 2500 grams including 22 (2.5%) < 1500 (very lbw) grams.

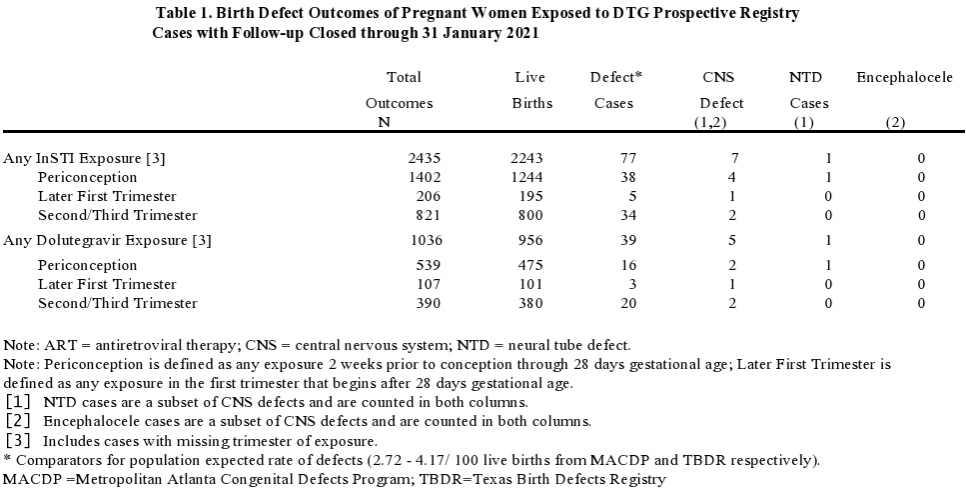

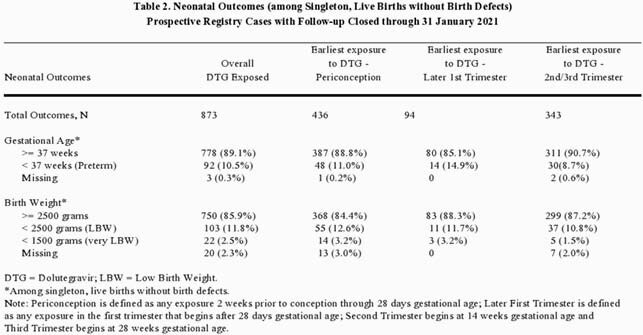

**Conclusion:**

APR data do not demonstrate an increased risk of overall birth defects with DTG use above the population expected rate of defects (2.72 to 4.17 per 100 live births from Metropolitan Atlanta Congenital Defects Program [MACDP] and Texas Birth Defects Registry [TBDR] respectively). The number of periconception exposure outcomes is not yet sufficient to evaluate potential association of DTG with NTD. The Registry continues to closely monitor birth defects, including NTDs in pregnancies exposed to DTG and other integrase inhibitors.

**Disclosures:**

**Vani Vannappagari, MBBS, MPH, PhD**, **ViiV Healthcare Limited** (Employee) **Jessica Albano, PhD, MPH**, **Syneos Health** (Employee, Shareholder) **Leigh Ragone, MS**, **GlaxoSmithKline** (Shareholder)**ViiV Healthcare** (Employee) **Taylor Cook, BS**, **Syneos Health** (Employee) **Angela Scheuerle, MD**, **ViiV** (Independent Contractor) **William R. Short, MD**, Gilead Sciences (Individual(s) Involved: Self): Consultant; ViiV (Individual(s) Involved: Self): Consultant **Claire Thorne, MSc, PhD**, **MSD** (Grant/Research Support)**ViiV Healthcare** (Grant/Research Support, Other Financial or Material Support, Contributor to Think Tank)

